# Retraction: Determinants of correct knowledge on tuberculosis transmission and self-reported tuberculosis prevalence among general population aged 15–49 years in Myanmar

**DOI:** 10.1371/journal.pone.0350447

**Published:** 2026-05-29

**Authors:** The PLOS One Editors

After this article [1] was published, concerns were raised about the methodology, results, and conclusions, and the article was re-assessed by multiple independent members of the *PLOS One* Editorial Board.

One of the consulting Editorial Board members raised concerns about the reported results and conclusions. Specifically:

1. The article [1] states that self-reported prevalence results were derived from the DHS survey question “Have you ever been told by a doctor/nurse or other health workers that you have/had tuberculosis?”, but a response to this question does not indicate current TB prevalence unless an associated time period is also reported.
2. Compared to the Variables subsection of the Methods section in [1], the responses to the question “How does Tuberculosis spread from one person to another?” in Table 2 includes two additional responses “Others” and “Don’t know”, and the article does not clarify whether participants could give one or multiple responses to this question.

In addition to the concerns above, the *PLOS One* Editors raised the following concerns:

1. The data underlying this study are derived from the Myanmar Demographic and Health Survey 2015–2016 report (DHS survey) [2]. The total number of participants of the DHS survey is 17,622. According to the report [2], this survey included the question “have you ever heard of an illness called tuberculosis or TB”. If the respondent answered “no” the follow-up questions “How does tuberculosis spread from one person to another?” and “Have you ever been told by a doctor/nurse or other health workers that you have/had tuberculosis” were skipped. The report also states that 93.2% of female participants and 92% of male participants had heard of TB, and lists the total number of respondents to the TB-related follow up questions as 12,012 and 4,357 for women and men respectively. However, the results in [1] analyzing the knowledge of TB transmission and the predictors of self-reported TB prevalence among the population reports analysis using the total number of respondents (n = 17,622), as opposed to the subsection of the respondents that answered further questions related to TB transmission, treatment, and prevalence (n = 16,369), suggesting that this analysis may also have included responses from individuals who, according to the survey report, were not subjected to these questions.
2. Upon editorial reassessment, it was noted that the data and parts of the results presented in this article are similar to results previously published in [2], raising concerns about the article’s adherence to the journal’s first criterium for publication [3].

Regarding point 1, the corresponding author stated that the use of the DHS survey as secondary data in [1] restricts the ability to capture detailed prevalence metrics, and that the survey question captures lifetime prevalence instead of current prevalence. The *PLOS One* Editors note that in paragraph nine of the Discussion section, the explanation given for the higher reporting of TB in the older age group includes that this may be due to older individuals having a weaker immune response system, but the Discussion section does not consider that the results reporting lifetime prevalence may be affected by the longer lifetime of subjects in the older age groups.

Regarding point 2, the corresponding author stated that in the original DHS questionnaire [2] analyzed in [1], multiple responses were allowed and that the question “How does tuberculosis spread from one person to another?” included the eight predefined response options listed in Table 2 of [1]. They stated that a rubric-based approach was applied to the six response options listed in the Variables subsection of the Methods section to construct the binary indicator of correct knowledge of TB transmission, and that these results are reported in Table 3. The corresponding author also stated that participants were classified as having correct knowledge only if they responded “Through the air when coughing or sneezing” and did not respond with any other responses listed in the Variables subsection of the Materials and Methods. Regarding the “Don’t know” option, the corresponding author stated this was classified as having incorrect knowledge, and the “Other” responses were rare and that participants selecting “Other” were likely to also select one or more of the remaining response options.

Regarding point 3 above, the corresponding author stated that they considered that individuals who have never heard of TB would not reasonably be expected to possess accurate knowledge regarding its mode of transmission, and therefore, in absence of subject responses to the follow-up questions, the authors classified participants who reported that they had not heard of TB as having incorrect knowledge of TB transmission. The *PLOS One* Editors remain concerned that the analysis of the results uses the total number of participants and included assumed responses for a subset of questions that were not answered by all participants, which calls into question the reliability and validity of the analysis.

In light of the above unresolved concerns which bring the reliability and validity of the results and conclusions into question, the *PLOS One* Editors retract this article [1].

PLA, KMW, and HMWM agreed with the retraction and apologize for the issues with the published article. KLS did not agree with the retraction and stands by the article’s findings.

In addition to issues above, concerns were raised about a potential competing interest between the authors and the member of the Editorial Board who handled the peer review for this article. PLOS regrets that this was not addressed prior to publication of the article.
